# HIF stabilization inhibits renal epithelial cell migration and is associated with cytoskeletal alterations

**DOI:** 10.1038/s41598-018-27918-9

**Published:** 2018-06-22

**Authors:** Simon Müller, Sonja Djudjaj, Janina Lange, Mihail Iacovescu, Margarete Goppelt-Struebe, Peter Boor

**Affiliations:** 1 Department of Nephrology and Hypertension, FAU Erlangen-Nürnberg, Erlangen, Germany; 2 Department of Physics, FAU Erlangen-Nürnberg, Erlangen, Germany; 3 Institute of Pathology, RWTH Aachen University, Aachen, Germany; 4 Department of Nephrology and Immunology, RWTH Aachen University, Aachen, Germany

## Abstract

Acute kidney injury (AKI) is a common and potentially lethal complication in the hospitalized patients, with hypoxic injury being as a major cause. The loss of renal tubular epithelial cells (TEC), one of the AKI hallmarks, is potentially followed by tubular regeneration process orchestrated by the remaining uninjured TECs that undergo proliferation and migration. In this study, we used human primary TEC to investigate the initiation of tubular cell migration and associated cytoskeletal alterations in response to pharmacological HIF stabilization which resembles the pathophysiology of hypoxia. Tubular cells have been shown to migrate as cohorts in a wound healing assay. Importantly, cells of distal tubular origin moved faster than those of proximal origin. HIF stabilization impaired TEC migration, which was confirmed by live single cell tracking. HIF stabilization significantly reduced tubular cell migration velocity and promoted cell spreading. In contrast to the control conditions, HIF stabilization induced actin filaments rearrangement and cell adhesion molecules including paxillin and focal adhesion kinase. Condensed bundling of keratin fibers was also observed, while the expression of different types of keratins, phosphorylation of keratin 18, and the microtubule structure were not altered. In summary, HIF stabilization reduced the ability of renal tubular cells to migrate and led to cytoskeleton reorganization. Our data suggested an important involvement of HIF stabilization during the epithelial migration underlying the mechanism of renal regeneration in response to AKI.

## Introduction

Acute kidney injury (AKI) is a common disease that affects up to 18% of all long-term hospitalized patients that increases the incidence of fetal clinical consequences^1^. Most AKI cases display proximal tubular cell injury and death resulting from renal hypoxia or ischemia and exposure to drug or toxin^2,3^. The renal tubular cells possess regenerative capacity that involves cell migration, proliferation and reconstitution of physiological functions^4^. Several studies have analysed the protective role of tubular cell proliferation during post AKI regeneration^5,6^, yet little is known about the role of tubular cell migration. After acute tubular cell injury and loss, a denuded basement membrane is rarely observed. This suggests a fast, initial migratory response is triggered in the remaining uninjured or sublethally injured cells to cover the exposed area of basement membrane after cell death, followed by the proliferative response in these migrated cells to repair the lesion^2,5^.

During tubular cell migration, the epithelial cells first lose their polarity and expand protrusions towards the direction of migration. These protrusions could be displayed as large, broad lamellipodia or spike-like filopodia and are often driven by actin polymerization^7^. Cytoskeletal rearrangement is an important process for cell motility and the involved proteins including F-actin stress fibers, microtubules or microfilaments such as vimentin have been largely studied. Some studies also indicated that the intermediate filament keratins are also involved in cell migration^8^. Like other simple epithelia, renal tubular epithelial cells (TECs) also express keratins. Keratin (K) is the largest subgroup of intermediate filaments and crucially involved in maintaining the structural integrity of epithelial cells^9^. Different types of keratins are expressed in an organ and epithelial cell-specific manner, of which K8, K18, K7 and K19 are the major keratins in the kidney. In our previous study, we demonstrated that keratin expression was upregulated with altered subcellular localization in various animal models and patients with overt renal tubular cell injury, including AKI. Therefore, keratins may serve as novel TEC stress markers for kidney disease^10^.

Hypoxia and ischemia are the well-known causes of tubular cell injury during AKI episode^3,11,12^. Several studies have shown the effect of hypoxia on cell migration, especially in cancer cells, but data on renal cells are rare^2,13,14^. The central signalling governing the hypoxic effects in cells involves the stabilization of hypoxia-inducible transcription factors (HIF). Pharmacologically, this can be achieved by inhibiting oxygen-sensing prolylhydroxylases (PHD) that prevents HIF degradation^15^. Dimethyloxalyl glycine (DMOG) is one of the commonly used PHD inhibitors for inducing HIF stabilization *in vitro*^16^. In the present study, we used an *in vitro* cell culture system of human primary tubular epithelial cells (hPTEC) to study the effects of DMOG treatment or hypoxia regarding the cell morphology and migration behaviour. We proposed a link between cytoskeletal reorganization during pharmacological HIF stabilization, such as bundling of keratin fibers and the reduced cell migration with enhanced cell spreading that might have implications in wound healing during AKI.

## Results

### DMOG reduces migration of tubular cells

**Figure 1:**
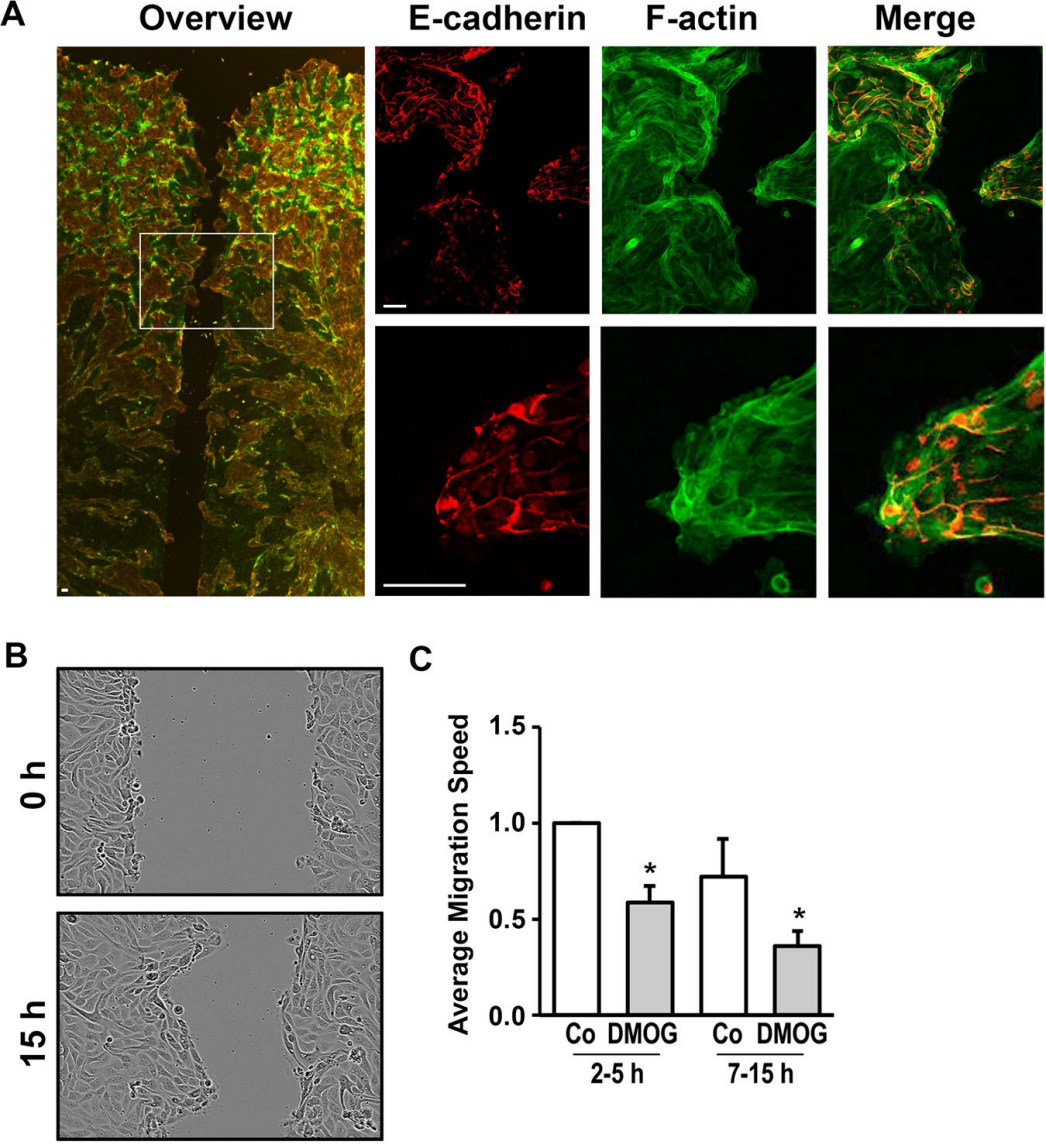
Migration of hPTECs was impaired by DMOG. (**A**) hPTECs were seeded in Ibidi barriers in 8-well slides and grown to confluence. After removal of the barriers, cells were allowed to migrate into the open space for 7 h. Cells were stained for E-cadherin (red) and F-actin (green). Scale bar: 30 µm. (**B**) Distal hPTECs were seeded as described above. Pictures of the wound were taken directly after removal of the barrier and 15 h later. (**C**) Distal hPTECs were seeded as described above. Cells were treated with DMOG (1 mM) 24 h prior to removal of the barriers. Movement of the cells was monitored using the program provided by the manufacturer (IncuCyte^R^, Essen Biosciences). Based on the confluency data, migration speed was calculated in arbitrary units per h, covering an early (2–5 h) and late (7–15 h) time-frame. In each experiment, migration of control cells between 2 and 5 h was set to 1. The graph summarizes data (means ± SD) of 3 experiments. *p < 0.05 compared to control cells.

Epithelial cells usually migrate as cohorts with intact cell-cell contacts. Therefore, we used the Ibidi migration barriers to obtain confluent monolayers which allowed the cells to migrate into a well-defined space. We first showed that the hPTEC migrated as a cohort. However, the migration occurred in a non-uniform pattern with irregular borders and large protrusions. This indicates an unequal rate of migration of various epithelial cell types (Fig. 1A).

The tubular cells isolated from healthy human kidney region of the tumor nephrectomies are derived from different parts of the renal tubular system. Roughly, they can be distinguished as proximal and distal tubule epithelial cells based on the expression of the cell-cell adhesion molecules namely N-cadherin and E-cadherin respectively^17,18^. The staining of the wound area showed that the fast-migrating and protrusion-forming cells were E-cadherin positive and therefore almost exclusively the distal tubular cells. The E-cadherin negative proximal tubular cells migrate slower and remained behind (Fig. 1A and Suppl. Figure 1).

Irregular migration behaviour was also observed in a pure *in vitro* culture of distal tubular cells (Fig. 1B). To determine the rate of cell migration, we measured the confluency of cells in the defined areas as shown in Fig. 1B, i.e. expressed as changes in confluency per hour. The change in confluency was monitored every 5 minutes and became linear after about 1–2 hours. Therefore, we chose two time slots, from 2 to 5 hours and from 7 to 15 hours, for the evaluation. Overall, we observed the migration rate declined over time (Fig. 1C). We postulated the declined migration rate was a result of the decreased compactness of the monolayer which is the driving force for cell migration. Compared to the unstimulated distal tubular cells, 24 hours treatment with DMOG (1 mM) before removal of the barriers resulted in a slower migration rate, especially between 7 and 15 hours post barrier removal (Fig. 1C). Migration can be influenced by inhibition of proliferation upon DMOG treatment. Therefore, we determined the total protein concentration as a surrogate marker for proliferation. After 24 hours of DMOG stimulation it was found to be 94 + 15%, DMOG versus control cells (n = 6 different preparations). DMOG-mediated effects on cell proliferation may partially contribute to the reduction in migration velocity, especially at the early time-point after removal of the barrier.

### DMOG impairs the movement of individual hPTEC

**Figure 2:**
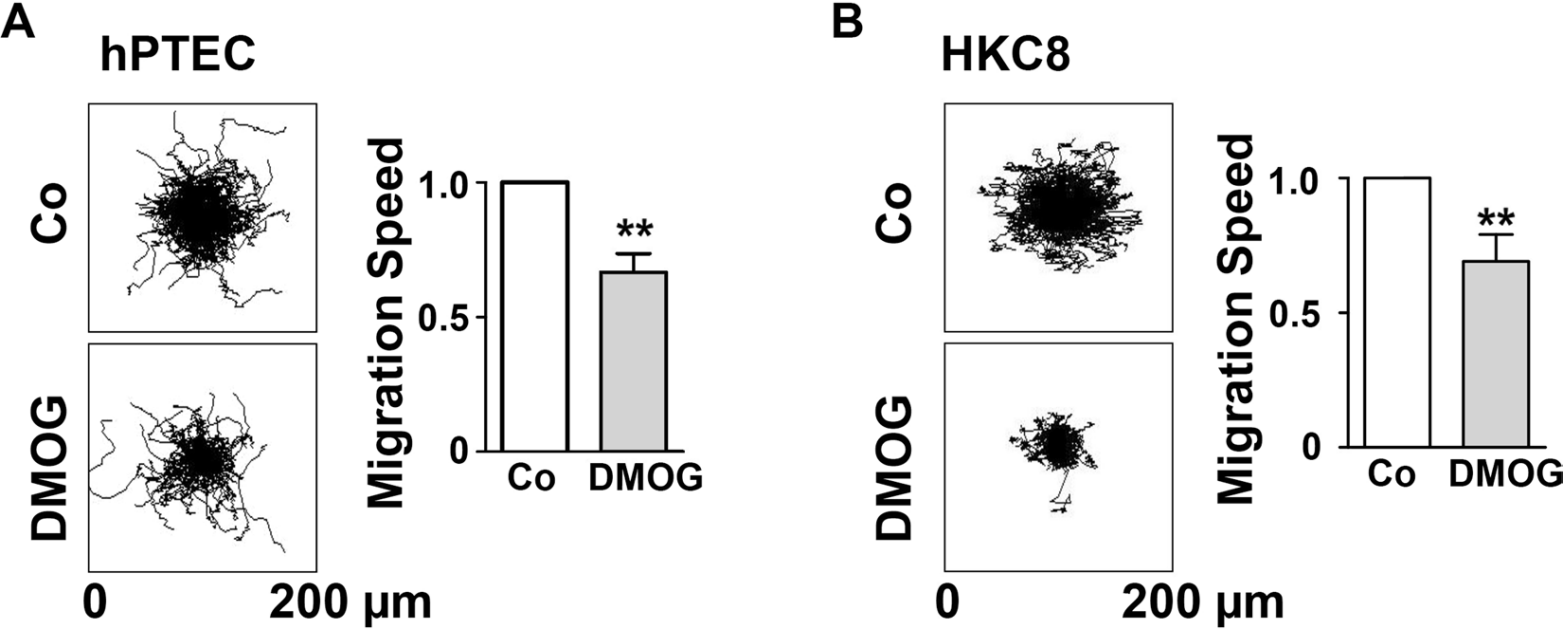
Migration velocity of single cells was reduced by DMOG. hPTECs (**A**) and HKC-8 cells (**B**) were incubated with DMOG for 24 h followed by labeling with HOECHST 33342 for 1 h. Cells were then seeded at low density (15 000 cells/cm^2^) and allowed to adhere for 2 h. To monitor cell migration, images were taken every 5 min. Representative star plots were depicted. Migration speed was determined in the time interval 2–5 h. Graphs summarize results of 3 experiments with two observation points each (means ± SD). **p < 0.01, one sample t-test.

To study the direct effect of DMOG on cell migration with exclusion of two confounders, cell death and cell proliferation, thereby we determined the cell migration of individual cells. hPTECs were treated with DMOG, then labelled with Hoechst 33343 and seeded fresh. Images of the hPTECs were taken every 5 minutes and analysed by Matlab. The migration was monitored for 5 h and the result was illustrated as a star plot in Fig. 2A. The motility of the individual cells was very heterogeneous, in accordance with the data obtained in the barrier assay described above. Treatment of cells with DMOG for 24 h prior to seeding reduced the cell motility as quantified by calculating the average migration rate (Fig. 2A). These data were reproduced in experiments with a human tubular epithelial cell line, HKC-8, the results were found to be less heterogeneous (Fig. 2B).

Taken together, these data demonstrated that inhibition of PHDs by DMOG results in slower migration of primary human tubular epithelial cells.

### DMOG leads to reorganization of keratin fibers

**Figure 3:**
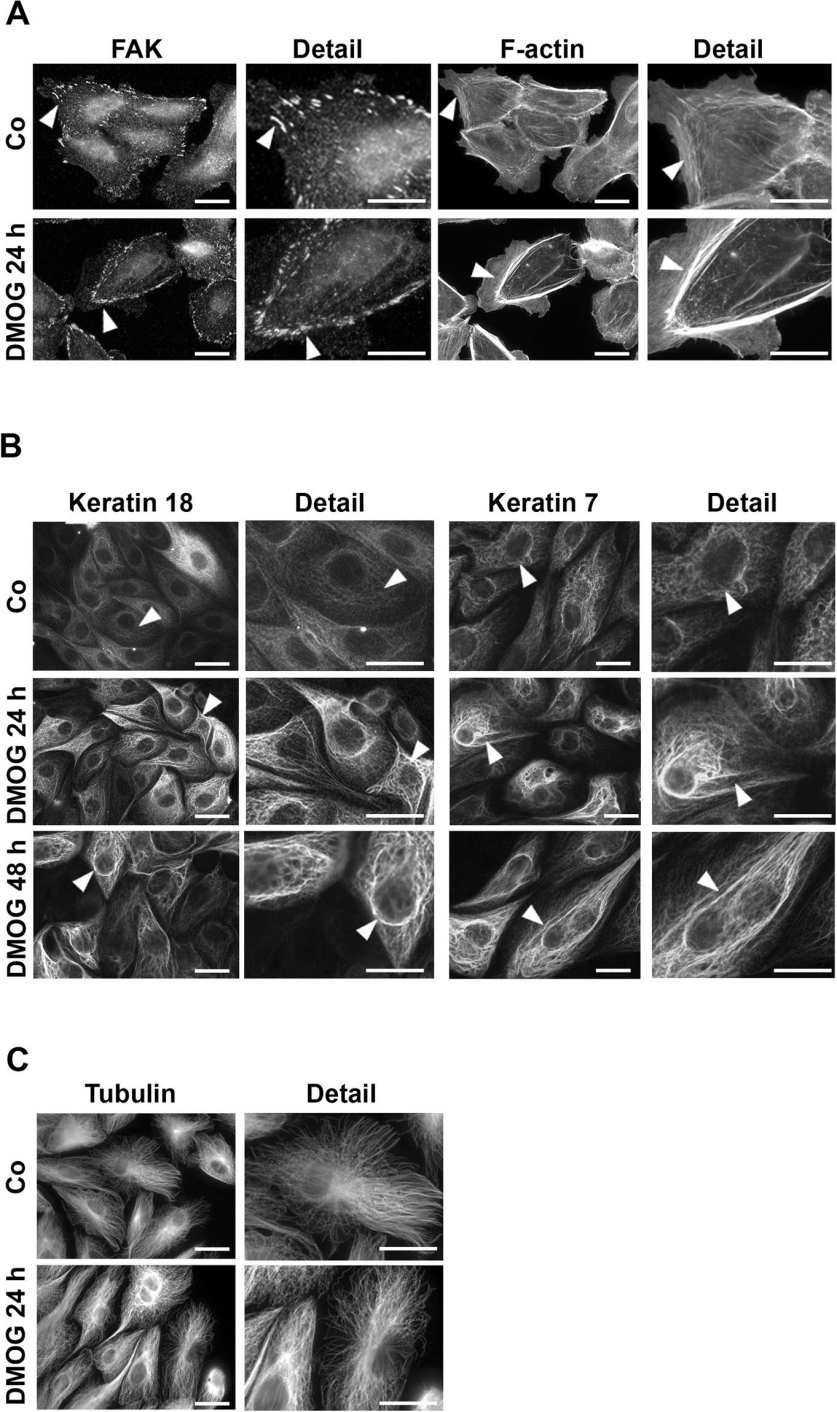
DMOG-induced structural changes in hPTECs. (**A**) Sub-confluent hPTECs were incubated with DMOG for 24 h. Focal adhesion kinase (FAK) and F-actin were detected by immunofluorescence. Scale bar: 10 µm. (**B**) hPTECs were treated with 1 mM DMOG for 24 or 48 h. Keratin 7 and 18 were visualized by indirect immunocytochemistry. Scale bar: 10 µm. (**C**) hPTECs were treated with DMOG for 24 h. Microtubuli were visualized by staining with anti-tubulin antibodies. Scale bar: 10 µm.

Next, we studied the changes in cell structure associated with migration. hPTECs were seeded on fibronectin-coated coverslips and analysed by indirect immunofluorescence after 24 h treatment of DMOG. Alterations in cell adherence were best detected at the boundaries of sub-confluent monolayers. Focal adhesions, which were visualized by staining of the focal adhesion kinase (FAK), were organized in irregular clusters within the control cells (arrowheads in Fig. 3A). In contrast, DMOG-treated hPTECs displayed smaller, well-aligned focal adhesions which were organized along the strong subcortical F-actin bundles (arrowheads in Fig. 3A). Similar data were obtained when focal adhesions were visualized by paxillin staining (Suppl. Figure 2). Such changes in focal adhesions were even more pronounced in spreading cells as described below. Thus, the impaired movement of hPTECs observed upon DMOG treatment could be reflected in the altered cytoskeletal structures.

Next, we studied another crucial component of renal tubular cell cytoskeleton, the intermediate filament keratins. We hypothesized that keratins might contribute to structural alterations in tubular epithelial cells and could represent as sensitive stress markers in TECs *in vivo*^10^. We focused our analyses on K18 which was one of the mostly regulated keratins in our previous studies in animals and humans with renal diseases. In cell culture, K18 was expressed mainly in the N-cadherin-negative distal hPTECs and only weakly expressed in the N-cadherin-positive proximal hPTECs (Suppl. Figure 3A). The K18 expression varied within the population of distal hPTEC as shown in Suppl. Figure 3B, consistent with the differential expression of K18 in individual tubules and each tubular cell as detected by immunohistochemistry (Suppl. Figure 3C). In confluent hPTECs, K18 formed a cell-spanning network of fine fibers (Fig. 3B, control). When the cells were treated with DMOG for 24 or 48 h, K18 fibers were reorganized into thick-appearing bundles and appeared more condensed around the nuclei after 48 hours (Fig. 3B arrowheads). The rearrangement of the keratin fiber network was not restricted to K18 and also observed with K7 (Fig. 3B arrowheads). Unlike the keratins, microtubular fibers were not restructured upon DMOG treatment (Fig. 3C).

### DMOG does not increase keratin protein expression

**Figure 4:**
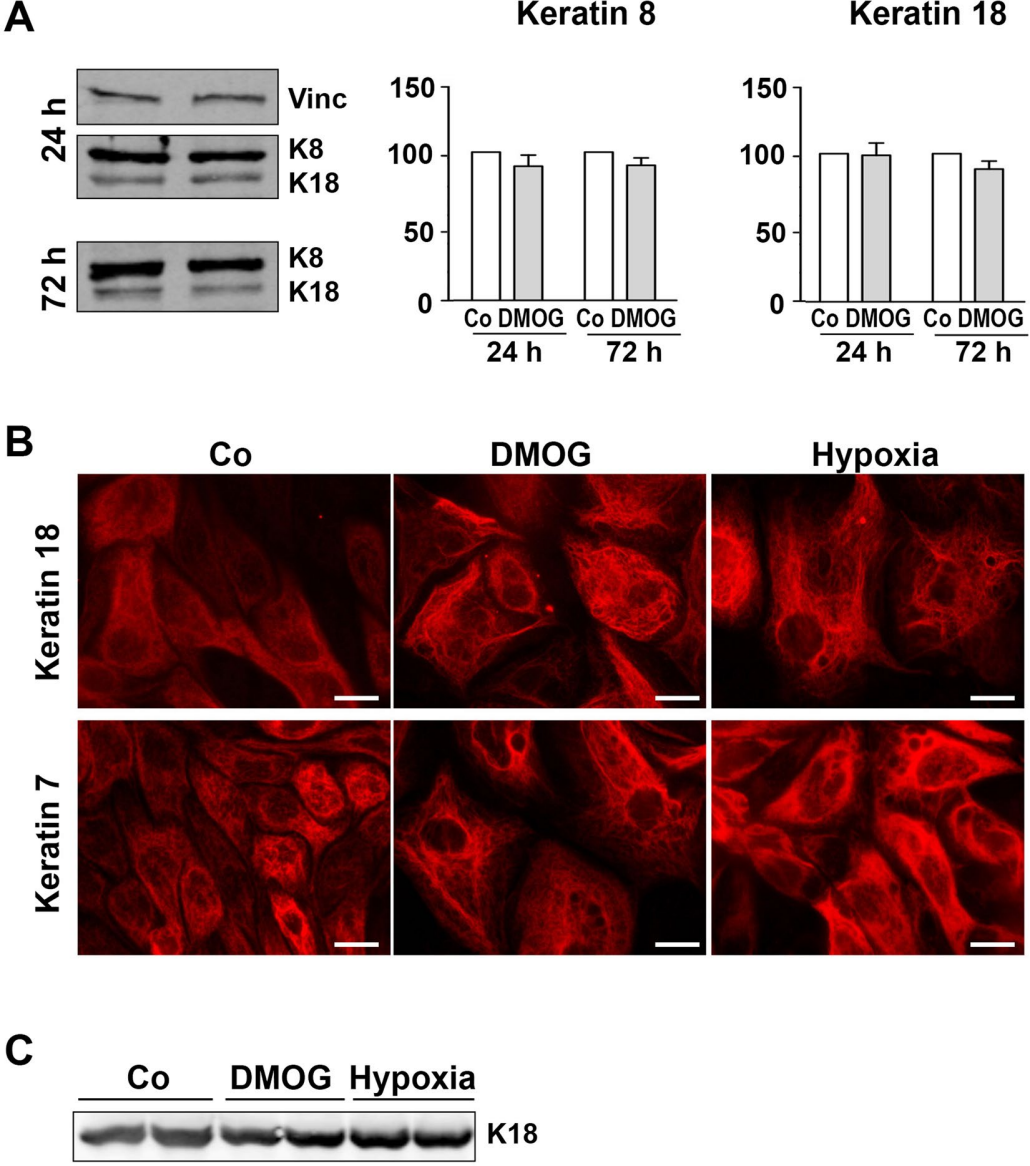
DMOG does not increase K18 expression in hPTECs. (**A**) hPTECs were incubated with DMOG (1 mM) for 24 h and 72 h. Expression of K18 and K8 was detected by Western blotting using a dual specific antibody. Blots from the 24 h experiment were reprobed for vinculin (Vinc). The graphs summarize data obtained by densitometric quantification of n = 3–4 experiments with different isolations (means ± SD). Data obtained with control cells were set to 100. (**B**) hPTECs were exposed to low oxygen pressure (1%) or treated with DMOG (1 mM) for 72 h. Keratin fibers were visualized by indirect immunofluorescence. Scale bar: 10 µm. (**C**) hPTECs were exposed to low oxygen pressure (1%) for 72 h. Expression of K18 in duplicate biological samples was shown in the representative Western blot image.

Next, we addressed the question whether structural changes in keratins are associated with the alterations in protein expression level. hPTECs were incubated with DMOG for up to 72 h. Keratin expression was assessed by Western blotting using an antibody that could detect both K18 and K8 (Fig. 4A). Incubation of cells with DMOG did not significantly alter the amount of K8 and K18 as quantified by the densitometries (graphs in Fig. 4A). Similarly, incubation with DMOG did not significantly modulate the amount of K7 or K19 synthesis in the hPTECs (Suppl. Figure 4).

Application of the PHD inhibitor DMOG in cell culture could closely recapitulate the hypoxic mechanisms, but its effects on cells are not exactly identical to the hypoxic exposure. Therefore, we also tested the effects of exposure of hPTEC to low oxygen pressure (1%). Expression of K18 after treatment with DMOG or exposure to hypoxia for 72 h is shown in Fig. 4B. Similar to DMOG treatment, no significant change in the amount of K18 protein was detectable (Fig. 4C), but a similar reorganization of the keratin network was observed (Fig. 4B). These data indicated that changes in keratin structure were mediated by the HIF activation. Additional effects of DMOG on other PHDs could not be excluded.

### Keratin 18 is phosphorylated in isolated tubular epithelial cells

**Figure 5:**
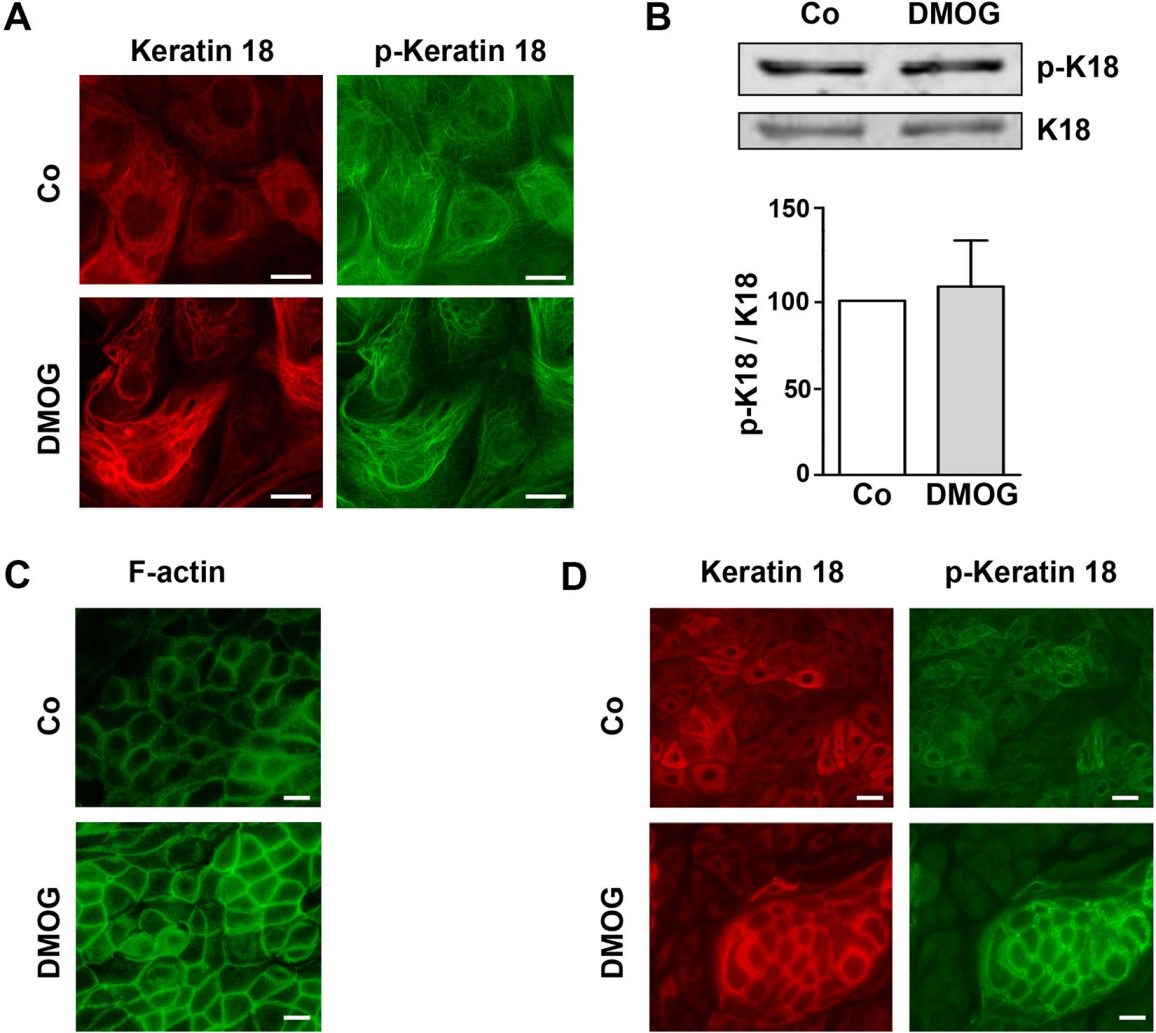
K18 was phosphorylated in hPTECs. (**A**) Confluent distal hPTECs were treated with DMOG for 24 h. K18 and phospho-K18 were detected with specific antibodies by indirect immunocytochemistry. Scale bar: 10 µm. (**B**) hPTECs were incubated with DMOG for 24 h. K18 and phospho-K18 were detected by Western blotting. The graph summarized data of 3 independent preparations (means + SD). (**C**) hPTECs were polarized by culturing in transwell inserts for 8 days and then treated with DMOG for 24 h. Cells were stained for F-actin using phalloidin. Scale bar: 10 µm. (**D**) Polarized hPTECs were incubated with DMOG for 72 h. K18 and phospho-K18 were detected by indirect immunofluorescence. Scale bar: 10 µm.

Keratin network organization during migration, cell division and apoptosis is closely linked to the protein phosphorylation level, and keratin phosphorylation is known to be involved during epithelial stress response^19^. In our isolated hPTECs, Ser-33-phosphorylated keratin 18 was detected in the unstimulated cells (Fig. 5). Treatment with DMOG on the isolated hPTECs did not affect the K18 phosphorylation status as shown by immunocytochemistry (Fig. 5A) or Western blotting (5B).

*In vivo*, renal tubular epithelial cells are highly polarized. To more closely resemble the *in vivo* cell physiologies, hPTECs were cultured in transwell inserts to allow the cells to be polarized as previously described^20^. The polarized cells were characterized by the regular copperstone-like pattern visualized by F-actin staining localized at the cell boundaries (Fig. 5C). Treatment of DMOG for 24 h further accentuated the F-actin fibers. In the polarized hPTECs, K18 fibers were organized in an irregular network within the cells (Fig. 5D). When the cells were treated with DMOG, the fibers appeared being more organized. They accentuated around but sparing the nuclei. In fact, we observed the K18 proteins were prominently phosphorylated at Ser-33 under these culture conditions (Fig. 5D).

### DMOG accelerates cell attachment *in vitro*

**Figure 6:**
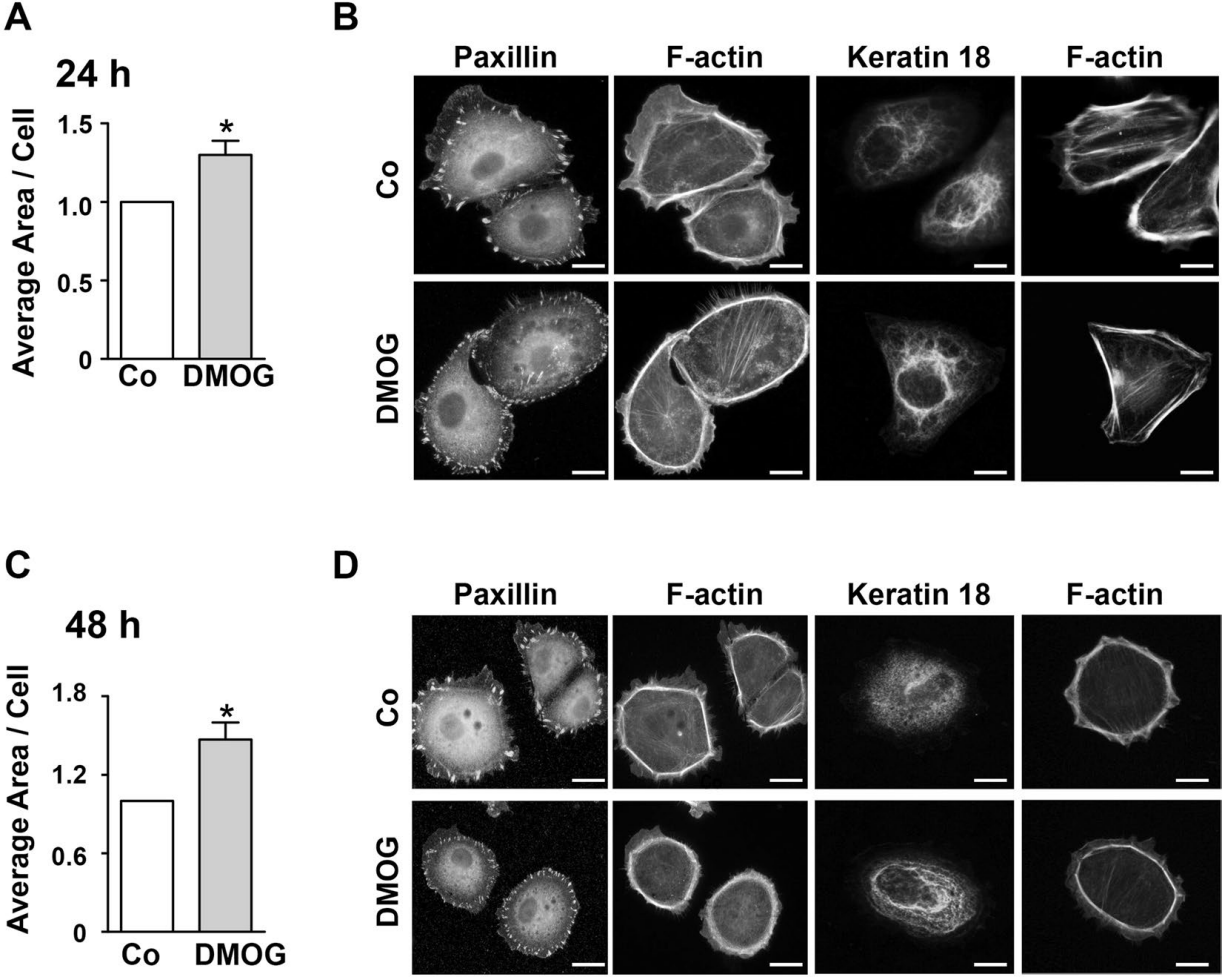
Spreading of hPTECs. (**A**) hPTECs were treated with DMOG for 24 h and then seeded at low density (15 000 cells/cm^2^) on fibronectin-coated glass cover slips. Spreading of hPTECs was assessed 2 h after seeding. Total area covered by phalloidin-stained cells was divided by the number of cells determined by nuclear staining with DAPI. In each experiment, 8–12 randomly chosen fields were evaluated. Data were expressed as means ± SEM of 4 independent experiments. *p < 0.05, one-sided t-test. (**B**) hPTECs were seeded as in A and treated with DMOG for 48 h. Data were expressed as means ± SEM of 4 independent experiments. *p < 0.05, one-sided t-test. (**C**) hPTECs were treated as in A. Paxillin and K18 were detected by indirect immunofluorescence and F-actin was visualized by phalloidin. Examples of spread cells were shown. Scale bar: 10 µm. (**D**) hPTECs were treated as in C. In the spread cells, paxillin and K18 were detected by indirect immunofluorescence and F-actin was visualized by phalloidin. Scale bar: 10 µm.

To better understand the impact of DMOG on hPTEC migration, the adhesive properties of hPTEC were investigated. After treatment of DMOG for 24 h, hPTECs were seeded at low density on the fibronectin-coated glass cover slips. 2 h after seeding, the expression of cell adhesion molecules, K18 and cell spreading were analysed. Not surprisingly, the state of adherence varied among the seeded cells. Some cells still appeared round in shape and others were well spread and adherent. The average area per cell was more pronounced in DMOG-treated cells compared to control cells at both 24 and 48 h post seeding (Fig. 6A,B) and this phenomenon was associated with changes in cytoskeletal structure (Fig. 6C,D). Typically, DMOG-treated cells showed strong cortical F-actin and well aligned focal adhesions compared to the stronger and separated focal adhesions of control cells shown with paxillin staining (Fig. 6C,D). A similar pattern was observed by FAK staining (data not shown). K18 formed a dense network of fibers in the control cells, whereas aggregated fibers which localized at perinuclear region were frequently observed in DMOG-treated cells (Fig. 6C,D). These data indicated that DMOG induced accelerated adhesion and spreading of hPTECs that was associated with the cytoskeletal remodelling.

## Discussion

Epithelial cell migration is an important process in tissue morphogenesis and repair after injury. In kidney diseases, damaged tubular epithelial cells exhibit an altered phenotype, which is also characterized by increasing tendency to replace cell loss as part of the repair process^2,5^. Reduced cell migration may therefore affect renal regeneration after injury. The major novel findings of our study are reduced cell migration of primary human tubular epithelial cells by pharmacological HIF stabilization that is associated with structural changes and reorganization of the keratin cytoskeleton, suggesting a potential mechanism of blunted epithelial wound healing during kidney injury.

In this study, we used a cell culture model with primary human renal tubular cells to examine the effect of HIF stabilization on wound healing and cell migration. Only a few studies have addressed the effects of hypoxia on tubular cell migration^2,14,21,22^. Compared to these studies, we performed our analyses in primary human renal tubular cells, currently one of the highly resemble human model systems. Treatment of cobaltous chloride (CoCl_2_) or low oxygen (1%) on rat proximal tubular cells (RPTCs) for 6–15 days has been reported to induce tubular cell dedifferentiation and increased cell migration^22^. Intriguingly, others reported that cell migration rate of RPTC is reduced during the first day of low oxygen (1%) stimulation^14^. This discrepancy could be explained by the intensity and duration of hypoxia, although the relationship between the severity of hypoxia and cell migration is not yet understood. In particular, the migration of tumor cells at borders is regulated differently by the duration of oxygen deprivation: short-term hypoxia with stabilized HIF inhibits cell migration, while extensive oxygen starvation stimulates cell migration^13^. Under hypoxic conditions, tubular epithelial cells isolated from mice adopted the mesenchymal phenotype that is characterized by the expression of alpha smooth muscle actin and migrated more rapidly^21^. In our study and other previous reports, exposure to low oxygen tension by stabilization of HIF did not induce a mesenchymal phenotype in human tubular epithelial cells^17^. Differences in species and experimental setup, e.g. the use of genetic vs. pharmacologic inhibition of HIF1 and of HIF1α^−/−^ epithelial cells in the context of hypoxia, may contribute to the different migratory behaviour of human vs. rodent tubular cells *in vitro*.

Epithelial cells retain cadherin-mediated cell-cell junctions during collective migration and assembly of cytoskeleton and cell-matrix junctions. Therefore, the dynamics of cytoskeletal arrangement is a key cellular mechanism for cell migration. It has been analysed mainly for F-actin stress fibers, microtubules or microfilaments like vimentin. Our study showed there were smaller focal adhesions, reduced lamellipodia and actin rearrangement in the hPTECs with HIF stabilization. This indicates stronger adherence to the substrate, which would explain the slower cell movement. For the first time, we examined the rearrangement of intermediate filaments, i.e. keratins in the migratory tubular epithelial cells that exposed to hypoxia or PHD inhibitor treatment. The structure of keratin fibers observed in tubular epithelial cells is in line with the previous reports of various epithelial cells *in vitro*, which shows fine filaments and stronger bundles in the periphery of nucleus^23,24^. In the hPTECs, exposure to hypoxia or treatment with DMOG increased thickness of keratin bundles around the nucleus. This finding is similar to the circumferential staining of keratins in kidney tissues of animals and patients with kidney diseases^10^. The altered keratin structures from fine filaments to bundles, namely bundling, is a complex reaction and typically independent of protein synthesis^24^. Our data confirmed this finding. Even a prolonged exposure to DMOG or hypoxia did not significantly alter the total protein amount of K8, K18, K7 and K19 in renal epithelial cells.

Keratin bundles have previously been described to strengthen and thereby protect cohorts of epithelial cells against traction forces, e.g. during migration. The role of keratins in collective epithelial migration has been previously studied mainly in the context of epidermal keratinocytes and skin wound healing. The findings are often obtained from a 2D cell culture system which is similar to our *in vitro* model^25–27^. Enhanced keratin bundling has been shown to cause delayed wound closure, while inhibition of keratin bundling or deletion of keratins leads to faster cell migration^28^. Our results demonstrated keratin bundling occurs under HIF stabilization and hypoxia. This could be directly involved in the observed reduction in wound healing, as keratins are directly connected to the cell-cell contact (desmosomes) and cell-adhesion contacts (hemidesmosomes). Some studies also observed an influence of keratin filaments on focal adhesion, but this mechanism has barely been studied^29^.

The bundling of keratins is regulated by post-translational modification, e.g. phosphorylation, but also by regulatory proteins^30^. In the alveolar epithelial cell, keratin bundling is associated with an increase in the phosphorylation of K8 (p-73) and K18 (p-33)^31,32^. Here we observed a strong phosphorylation of K18 (p-33) under basal conditions which was not altered during DMOG treatment and was detectable in both fine and bundled fibers. Whether other K18 residues that will undergo phosphorylation (e.g. p-52)^30^ or other post-translational modifications in renal tubular cells upon hypoxia or during migration needs further studies.

In the polarized epithelial cells, DMOG induced a strong pattern of F-actin at the cell boundaries and the keratins appeared to be patterned inside the cell. Similar structural alterations of F-actin upon DMOG treatment were previously observed in endothelial cells that were organized into three dimensional spheroids and associated with increased cell-cell adhesion^33^.

We have previously shown that TGF-β stimulation caused the cells obtained from proximal tubules to rearrange their cell structure towards a mesenchymal-like and thus adopted more migrating phenotype compared to cells derived from distal tubules^18^. It was therefore surprising that E-cadherin positive cohorts of cells, i.e. cells of distal tubular origin, migrated faster than proximal cells. This may be related to their ability to form stronger cell-cell adhesions which remains stable even upon TGF-β treatment^34^ thus favouring collective cell migration. Collective cell migration is well described for cancer cells^35,36^, in morphogenesis^37^ and in wound closure^28,38^. Here we showed that primary human tubular epithelial cells could also migrate as a cohort, suggesting that this process might be a highly conserved mechanism for wound closure, promoting re-epithelialisation and regeneration. Our results are also consistent with recent *in vivo* imaging studies in zebrafish, showing that collective cell migration is a driving force behind kidney epithelial repair and tubule resealing after acute injury^2^.

We have tried to manipulate K18 expression in our human primary TECs. However, we were unable to achieve sufficient knock-down of K18 protein expression, most likely due to the K18 turn-over time and stability. Our conclusions on the potential role of keratin cytoskeleton on cell migration rely therefore on descriptive correlations which is a limitation of this study. Another limitation is the pathological mechanisms that take place in AKI *in vivo* model are more complex compared to our *in vitro* model. Particularly, hypoxia is often followed by reperfusion and normoxia *in vivo*, which is believed to be associated with HIF degradation and in parallel with the recovery process including migration. Although there are limited approaches to study cell migration *in vivo*, our data should be confirmed in future by *in vivo* studies.

In conclusion, our data showed that HIF stabilization reduced the migration of tubular epithelial which is associated with keratin bundling and increased focal adhesions. Manipulation of the migration characteristics of tubular cells could be considered as a potential target for improving renal regeneration.

## Materials and Methods

### Cell culture

Human primary tubular epithelial cells (hPTECs) were isolated from renal cortical tissues collected from healthy parts of tumor nephrectomies. The isolation of human cells was approved by the local ethics committee at the FAU Erlangen-Nürnberg and the consent of all donors was obtained. Briefly, the tissue was minced on ice, digested by DNAse I (Roche Diagnostics) and collagenase II (Gibco) and then sieved through 100 and 70 µm filters. Cells were cultured in DMEM/Ham’s F-12 supplemented with 10% fetal calf serum, 2  mM L-glutamine, 100  U ml^−1^ penicillin and 100 μg ml^−1^ streptomycin, 5 μg ml^−1^ insulin, 5 μg ml^−1^ transferrin, 5 ng ml^−1^ selenium (Sigma), tri-jodothyronin (T3) 10 ng ml^−1^, hydrocortisone 1 mg ml^−1^, and epidermal growth factor 100 μg ml^−1^ (Peprotech). After about 1 week, a culture of epithelial cells was obtained which could be positive for either E- or N-cadherin staining^17,18^.

For the following experiments, hPTECs were seeded in the medium containing 2.5% FCS to facilitate cell attachment. After 24 h, the medium was replaced by the FCS-free epithelial cell selective medium. Cells were then either stimulated with dimethyloxalyl glycine (DMOG) (Cayman Chemical) or exposed to low oxygen pressure (1% oxygen). Cells originated from proximal and distal tubules were separated by their differential adhesion to cell culture plastic. Trypsinization for 3 min resulted in a culture enriched with the proximal tubular cells (approximately 60% N-cadherin positive cells), while the remaining cells were over 90% E-cadherin positive, representing cells of distal tubular origin. Data of both cell types were combined if no significant difference was found between the cell populations. Distal hPTECs showed polarization by culturing them on the permeable transwell inserts (Millicell PCF, Millipore) for 10 days as previously described^17,28^. In all experiments, hPTECs were used in passages 1–3.

HKC-8 cells were kindly provided by L. Racusen (Baltimore, MD)^39^. Cells were recloned by limited dilution and cultured as previously described^17^.

### Migration assays

hPTECs were seeded into the silicone culture inserts placed in ibidi 8 well µ-slides. When the cells reached confluence, the inserts were removed so the cells could migrate into the open space. The migration was monitored in three areas in each well. Confluence was analysed using the IncuCyte^R^ live cell analysis system (Essen Biosciences) which measured the open (cell-free) space every 5 min.

Single cell migration was monitored by HOECHST-labeled nuclei. Cells were stained with HOECHST 33342 for 30 min prior to harvesting and reseeding. Images were taken every 5 min with a Keyence BZ9000 microscope. Cell movement was analysed by MatLab. For this purpose, the center of each fluorescent nucleus was traced by a centroid-algorithm in all consecutive fluorescence images. Cell trajectories were then reconstructed by a maximum-likelihood-algorithm. Cell velocity was calculated as the average distance covered by a cell between two consecutive images: $\langle v\rangle$ = $\langle \frac{\triangle x}{\triangle t}\rangle$. All values were normalized by the value of the control population in each plot.

### Cell spreading assay

hPTECs were grown to confluency and treated with DMOG for 48 h. The cells were then seeded at low density on fibronectin-coated glass cover slips. After 2 h, cells were washed carefully and PFA-fixed to analyze the expression of cell adhesion molecules K18 and the spread of the cells. In each well 8–12 fields were selected to quantify cell spreading. The area covered by F-actin-stained cells or the number of DAPI-stained nuclei was determined by ImageJ software to calculate the average cell size.

### Western blot analysis

Western blot analyses were performed essentially as previously described^40^ with the following antibodies: Rabbit monoclonal antibodies: anti-keratin 8 (Clone EP1628Y) and anti-keratin 18 (Clone E431–1), mouse monoclonal antibodies: anti-keratin 8/18 (Clone 5D3), anti-keratin 19 (clone Ks19.1), anti-keratin 7 (clone OV-TL 12/30) (Thermo Scientific), anti-phospho-keratin 18 (S33) (STJ91019) (St. John’s Laboratory, London); goat anti-keratin 19 (SC-3311) (Santa Cruz), mouse anti-vinculin (clone hVIN-1) from Novusbio. The immunoreactive bands were quantified by densitometry using the luminescent image analyzer (LAS-1000 Image Analyzer, Fujifilm, Germany) and AIDA 4.15 image analyzer software (Raytest) or Odyssey infrared imaging system (Li-Cor, Biosciences). For quantification purposes the intensity of specific bands was relative to the intensity of vinculin as a housekeeping control. However, treatment with DMOG or exposure to hypoxia for 72 h altered the expression of vinculin and also other housekeeping proteins such as HSP90. Therefore, the intensity of the specific bands was normalized to protein load. Cellular protein determined after 72 h reflected the cell numbers: the protein concentration was determined in 3 different preparations and relative to cell numbers: control 1, DMOG-treated cells 1.09 + 0.7. To compare the different blots, fold change of induction was calculated as indicated in the legends. All data are presented as mean + SD of n different experiments with cells obtained from at least 3 different donors.

### Immunocytochemistry

Cells were fixed with paraformaldehyde (3.5% in PBS) for 10 min and then permeabilized with 0.5% Triton X-100 in PBS for 10 min. After washing three times with PBS, cells were blocked with 1% BSA in PBS for 1 h at room temperature and washed once. Tissue sections were fixed with paraformaldehyde. After deparaffinization, sections were incubated with citrate buffer (10 mM) for 10 min in a microwave oven. Primary antibodies include mouse anti-keratin 18 (clone Ks18.04) (Progen), mouse anti-keratin 7 (clone OV-TL 12/30) (DAKO), mouse anti-E-cadherin (ab1416) (Abcam), rabbit anti-N-cadherin (SC-7939) (Santa Cruz), mouse anti-tubulin (clone D66) (Sigma-Aldrich); mouse anti-paxillin (clone 349/Pax) and mouse anti-FAK (focal adhesion kinase) (clone 77/FAK) (BD Biosciences). Secondary antibodies (PromoFluor Fluor® anti-mouse or anti-rabbit) were purchased from Promokine. F-actin was stained with PromoFluor 488 or 555 phalloidin (Promokine). After mounting, slides were viewed using a Nikon Eclipse 80i fluorescent microscope and digital images were taken with Visitron Systems 7.4 Slider camera (Diagnostic Instruments) using Spot Advanced software (Diagnostic Instruments) or Keyence fluorescence microscope BZ9000. The full focus function was used to visualize keratin fibers in different planes of the cell.

### Statistics

N Numbers refer to different experiments with cells obtained from at least three different donors. All values are presented as means + SD or means + SEM, as appropriate. Multiple samples were compared by ANOVA with an appropriate post hoc test. Two groups of samples were compared by t-test as indicated.

Requests for data, information or materials should be addressed to the corresponding author.

## Electronic supplementary material

Supplement
