# Supplementary material for: HIF stabilization inhibits renal epithelial cell migration and is associated with cytoskeletal alterations
Source: Sci Rep. 2018 Jun 22;8:9497. doi: 10.1038/s41598-018-27918-9 (PMC6015081; doi:10.1038/s41598-018-27918-9)

Supplementary data

HIF stabilization inhibits renal epithelial cell migration and is associated with cytoskeletal alterations

Simon Müller[1]*, Sonja Djudjaj[3]*, Janina Lange[2], Mihail Iacovescu[1], Margarete Goppelt-Struebe[1], Peter Boor[§3,4]

Supplementary Figures

Supplementary Fig. S1: Faster Migration of E-cadherin-positive distal tubules

hPTEC were seeded in Ibidi barriers in 8-well slides and grown to confluence. After removal of the barriers, cells were allowed to migrate into the open space for 7 h. Cells were stained for E-cadherin (red) and N-cadherin (green). Arrrows point to faster migrating cells. Arrowheads marked E- and N-cadherin positive cells. Scale bar: 40 µm

Supplementary Fig. S2: DMOG-induced morphological alterations.

Sub-confluent hPTEC were incubated with DMOG (1 mM) for 24 h. Paxillin and F-actin were detected by immunofluorescence. Scale bar: 10 µm

Supplementary Fig. S3: Variable expression of K18 in renal epithelial cells

A: Monolayers of hPTEC were stained with antibodies against N-cadherin and K18. Scale bar: 30 µm

B: K18 was detected in a monolayer of distal tubular cells. Scale bar: 30 µm

C: K18 was detected in human kidney sections by immunohistochemistry. Scale bar: 30µm

Supplementary Fig. S4: DMOG does not alter expression of K7 or K19 in hPTEC

hPTEC were incubated with DMOG (1 mM) for 24 h and 72 h. Expression of K7 and K19 was detected by Western blotting using specific antibodies. Blots obtained from cells incubated for 24 h were reprobed with Vinculin (Vinc). The graphs summarize data obtained by densitometric quantification of n=3 experiments with different isolations (means ± SD). Data obtained with control cells were set to 100.

Supplementary Fig. S5: Original blots

All blots used for the manuscript figures are shown without any modifications.

Supplementary Figures

Supplementary Fig. S1: Faster Migration of E-cadherin-positive distal tubules

hPTEC were seeded in Ibidi barriers in 8-well slides and grown to confluence. After removal of the barriers, cells were allowed to migrate into the open space for 7 h. Cells were stained for E-cadherin (red) and N-cadherin (green). Arrrows point to faster migrating cells. Arrowheads marked E- and N-cadherin positive cells. Scale bar: 40 µm

Supplementary Fig. S2: DMOG-induced morphological alterations.

Sub-confluent hPTEC were incubated with DMOG (1 mM) for 24 h. Paxillin and F-actin were detected by immunofluorescence. Scale bar: 10 µm

Supplementary Fig. S3: Variable expression of K18 in renal epithelial cells

A: Monolayers of hPTEC were stained with antibodies against N-cadherin and K18. Scale bar: 30 µm

B: K18 was detected in a monolayer of distal tubular cells. Scale bar: 30 µm

C: K18 was detected in human kidney sections by immunohistochemistry. Scale bar: 30µm

Supplementary Fig. S4: DMOG does not alter expression of K7 or K19 in hPTEC

hPTEC were incubated with DMOG (1 mM) for 24 h and 72 h. Expression of K7 and K19 was detected by Western blotting using specific antibodies. Blots obtained from cells incubated for 24 h were reprobed with Vinculin (Vinc). The graphs summarize data obtained by densitometric quantification of n=3 experiments with different isolations (means ± SD). Data obtained with control cells were set to 100.

Supplementary Fig. S5: Original blots

All blots used for the manuscript figures are shown without any modifications.

Supplementary Figure 1

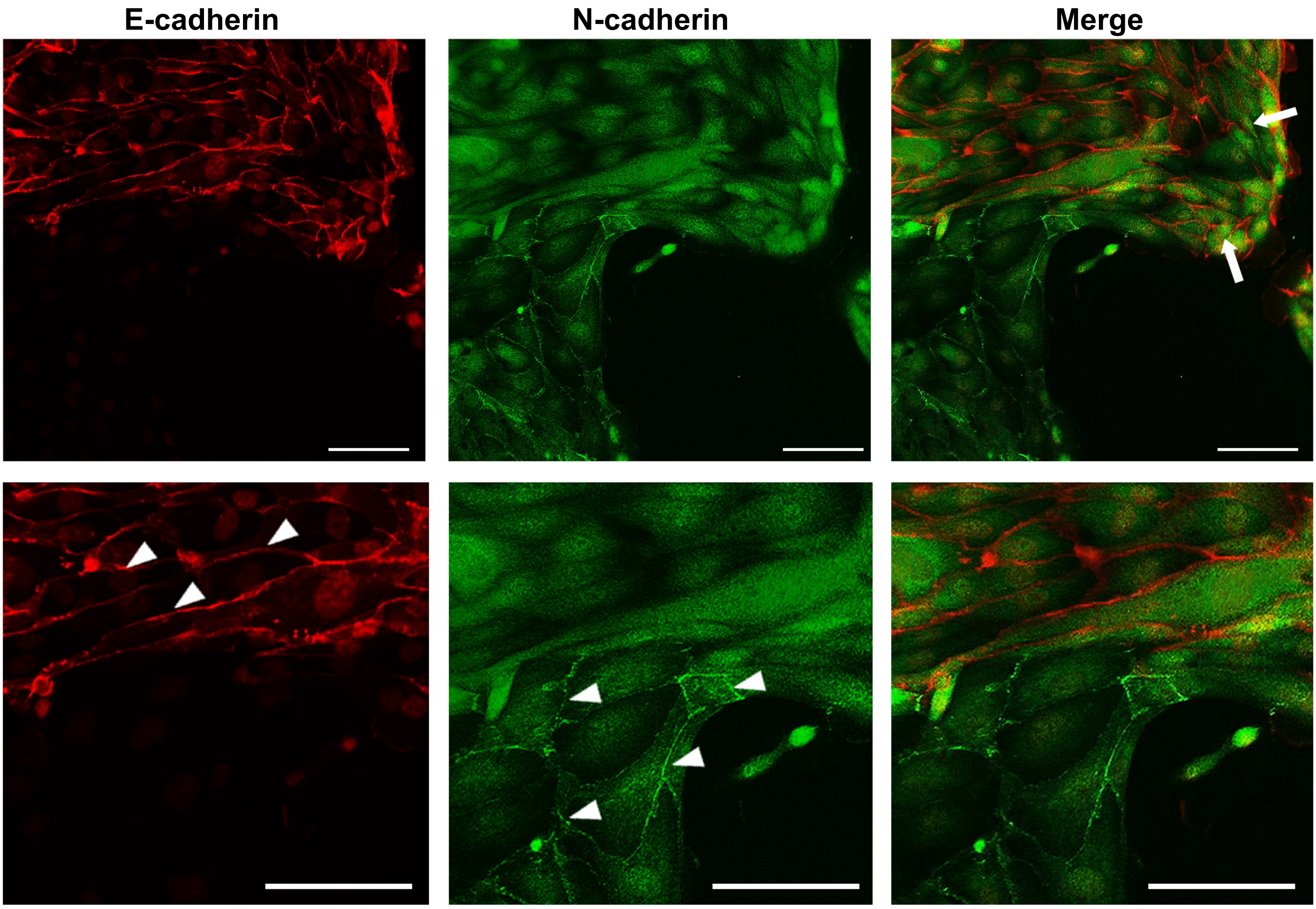

Supplementary Figure 2

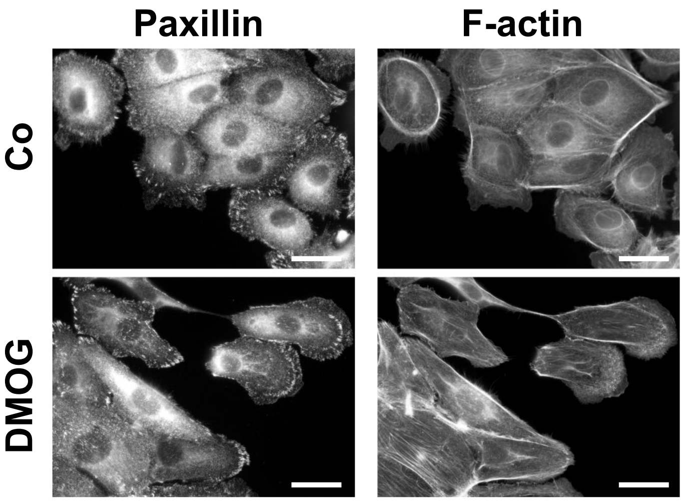

Supplementary Figure 3

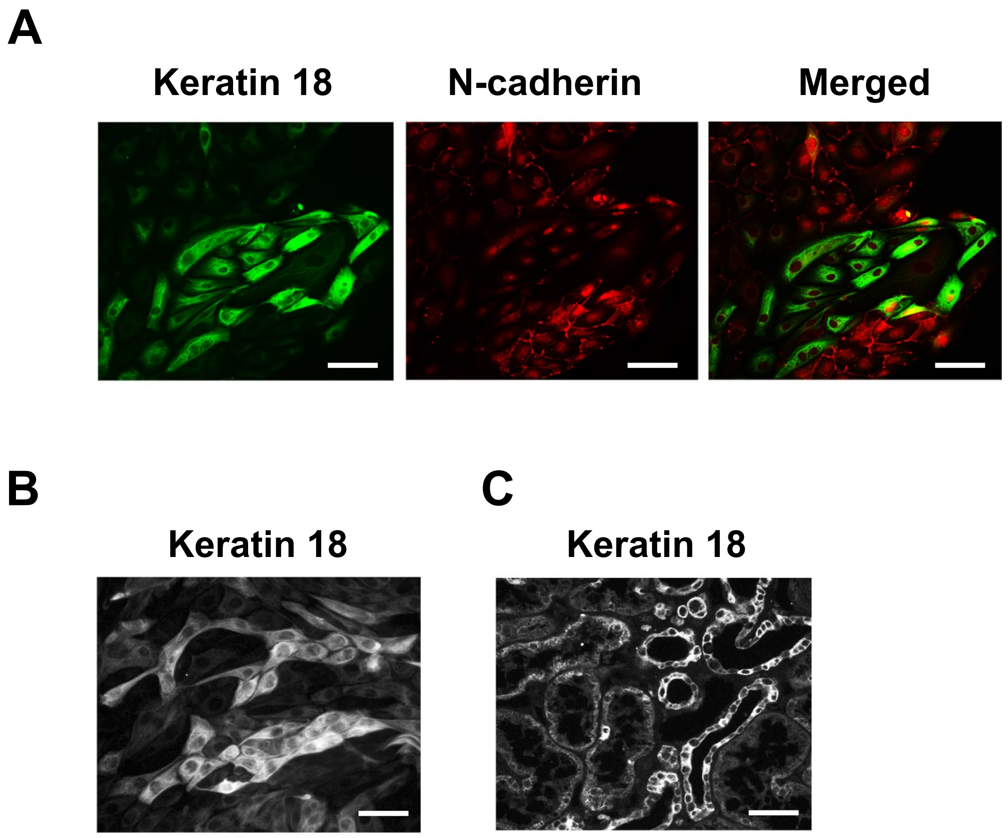

Supplementary Figure 4

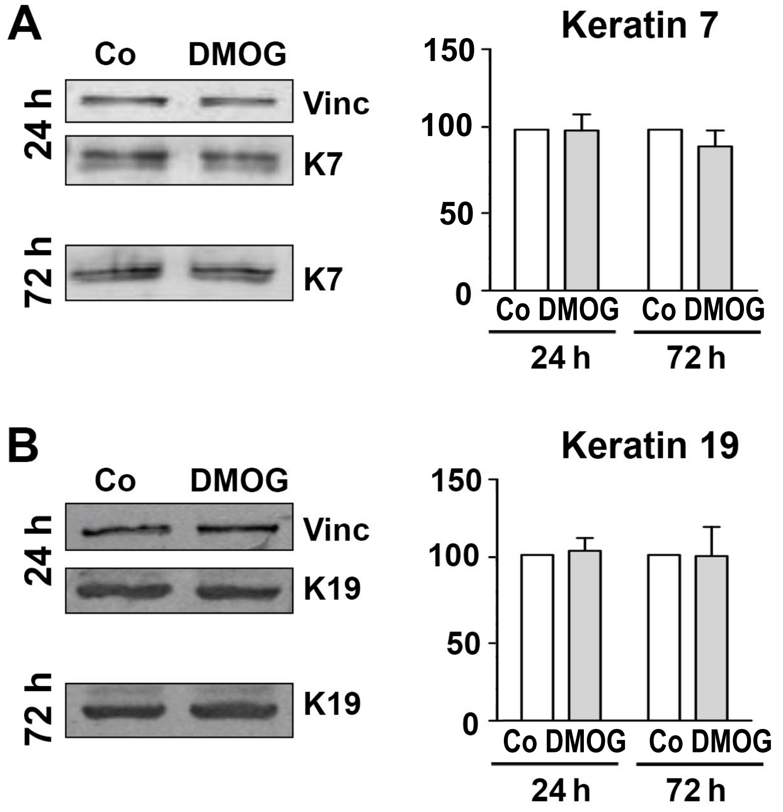

Supplementary Figure 5

Blots from Figure 4 B

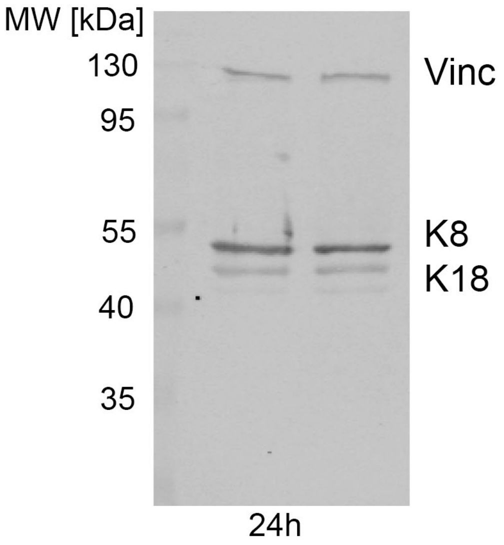

Blot from Figure 4 C

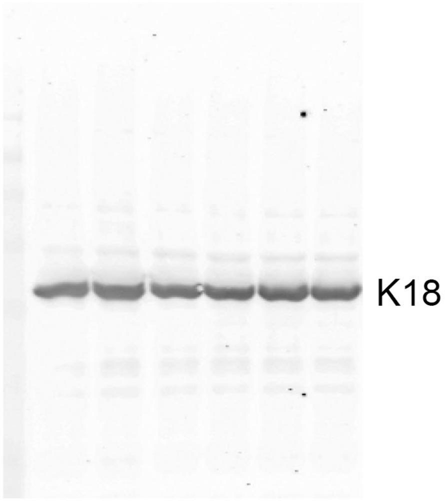

Blots from Figure 5 B

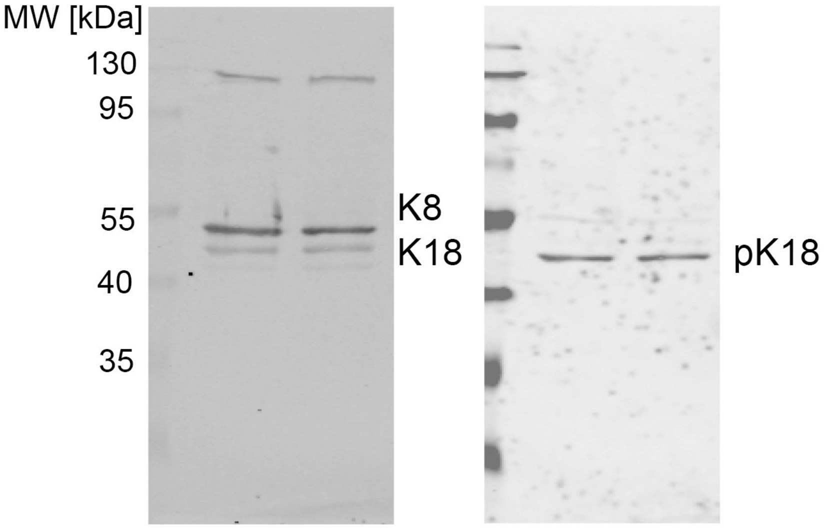

Blots from Suppl. Figure 4 A

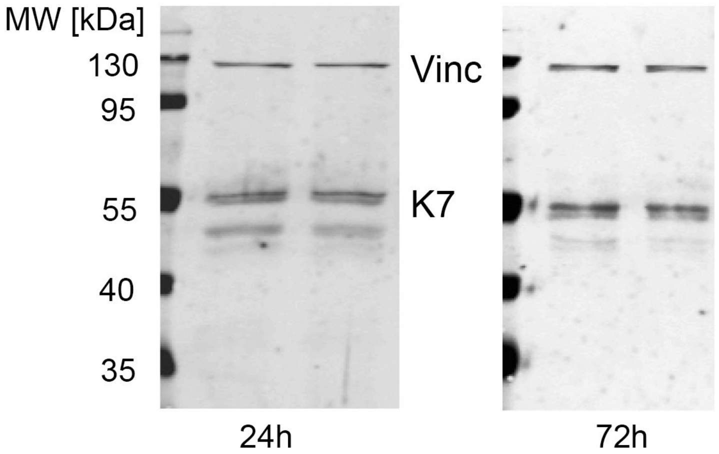

Blots from Suppl. Figure 4 B

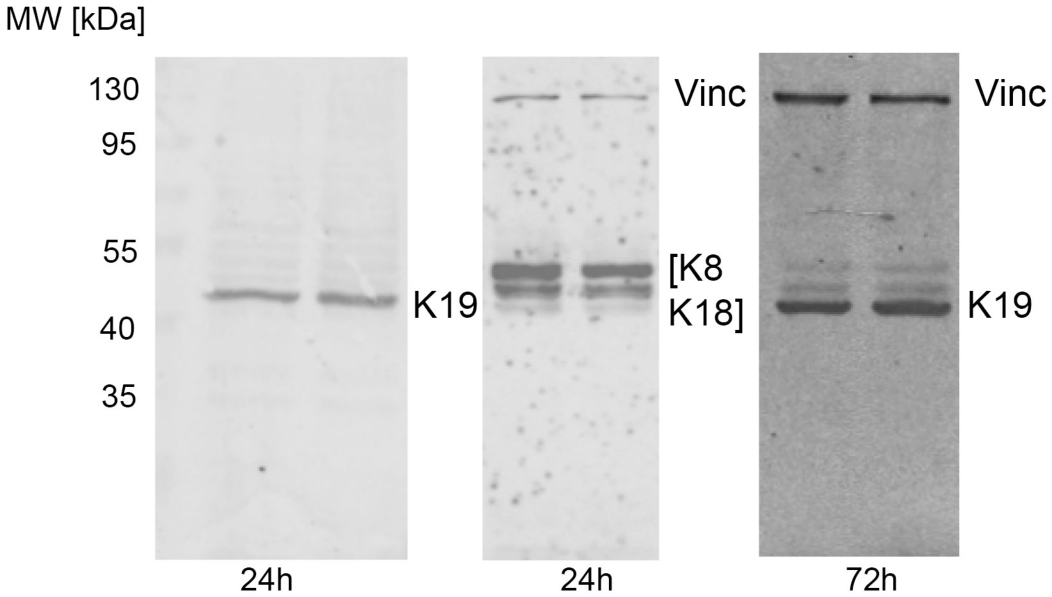

Supplement: Supplementary file 1 — Supplement [file 41598_2018_27918_MOESM1_ESM.pdf]
